# Electrical control of single-photon emission in highly charged individual colloidal quantum dots

**DOI:** 10.1126/sciadv.abb1821

**Published:** 2020-09-18

**Authors:** Sergii Morozov, Evangelina L. Pensa, Ali Hossain Khan, Anatolii Polovitsyn, Emiliano Cortés, Stefan A. Maier, Stefano Vezzoli, Iwan Moreels, Riccardo Sapienza

**Affiliations:** 1 The Blackett Laboratory, Department of Physics, Imperial College London, London SW7 2BW, UK.; 2 Department of Chemistry, Ghent University, Krijgslaan 281-S3, Gent 9000, Belgium.; 3 Chair in Hybrid Nanosystems, Nano-Institute Munich, Faculty of Physics, Ludwig-Maxilimians-Universität München, München 80539, Germany.

## Abstract

Electron transfer to an individual quantum dot promotes the formation of charged excitons with enhanced recombination pathways and reduced lifetimes. Excitons with only one or two extra charges have been observed and exploited for very efficient lasing or single–quantum dot light-emitting diodes. Here, by room-temperature time-resolved experiments on individual giant-shell CdSe/CdS quantum dots, we show the electrochemical formation of highly charged excitons containing more than 12 electrons and 1 hole. We report the control over intensity blinking, along with a deterministic manipulation of quantum dot photodynamics, with an observed 210-fold increase in the decay rate, accompanied by 12-fold decrease in the emission intensity, while preserving single-photon emission characteristics. These results pave the way for deterministic control over the charge state, and room-temperature decay rate engineering for colloidal quantum dot–based classical and quantum communication technologies.

## Teaser

The rate of photon emission from a quantum dot can be controlled and speed up by over hundred times by applying a voltage.

## INTRODUCTION

The observation of reduced Auger recombination, leading to an increased quantum yield of colloidal quantum dots, has sparked a fast-paced progress in the development of highly fluorescent and stable quantum dots for displays (*1*), light-emitting diodes (LEDs) (*2*), and coherent (*3*) and quantum light sources (*4*, *5*). Still, in the colloidal quantum dot community, there is an ongoing struggle to reconcile suppressed Auger recombination with fast radiative recombination (*6*). Existing systems that reduce Auger recombination also tend to have a lower electron-hole overlap (*6*) and therefore, increased fluorescence lifetime [surpassing 100 ns (*7*, *8*)], which hampers quantum and classical photonic technologies that rely on high brightness and fast communication rates.

A nanophotonic approach can boost light-matter interactions and modify an emitter’s decay rate by several orders of magnitude (*9*–*11*). Nevertheless, experimental studies have been limited so far to decay rate enhancements of ∼6 for a quantum dot surrounded by a plasmonic shell (*7*) or ∼80 inside plasmonic nanogaps (*12*) and patch antennas (*13*). This enhancement comes at the cost of a reduced single-photon emission purity due to strong biexciton emission, limited tunability, and the fabrication challenge of nanometric precision in positioning the quantum dots (*9*, *14*).

A different route is to exploit exciton charging to enhance the emission rate of quantum dots themselves, which can be realized by electrochemical (*15*–*18*) or photochemical (*19*, *20*) charge injection. Excitons with only one or two extra charges have allowed for the development of very efficient quantum dot lasing (*3*) and the understanding of blinking dynamics (*15*), while charge transfer management has yielded single–quantum dot LEDs (*4*), LEDs with reduced efficiency roll-off (*2*), and enabled studies of carrier and spin dynamics (*21*). The additional charge brings new recombination pathways—thus faster decay rates—and modifies the electronic state of the quantum dot due to Coulomb interactions, which are enhanced by strong spatial confinement and reduced dielectric screening (*22*).

Electrochemical injection of up to eight electrons in 1S*_e_* and 1P*_e_* states has been reported for thin ZnO (*23*), CdTe (*24*), PbSe (*25*, *26*), and CdSe (*26*–*29*) quantum dot films. In these cases, charge injection in the lowest quantum state has been verified by a bleaching of the ground-state exciton absorption. Extension of these electrochemical charging experiments to individual quantum dots, beyond ensemble averaging, has been hampered by sample degradation at high voltages and poor photostability of the quantum dots. This has been remedied by exploiting the giant-shell quantum dot architecture, whereby different emissive quantum states have been resolved in doubly charged CdSe/CdS and CdSeS/ZnS quantum dots, showing a reduced blinking and a modulated photoluminescence intensity and lifetime (*15*, *17*, *18*, *30*).

Here, we go beyond the weak charge injection regime, and we report the observation of controllable, stable, and highly charged excitonic states in an individual giant-shell CdSe/CdS quantum dot. To induce the highly charged states, we used a lithography-free electrochemical cell. We show reversible control of individual quantum dot single-photon dynamics, allowing for an on-demand increase in spontaneous emission decay rate up to 210-fold, with only a minor 12-fold decrease in emission intensity.

## RESULTS

### Quantum dots and experimental setup

**Fig. 1:**
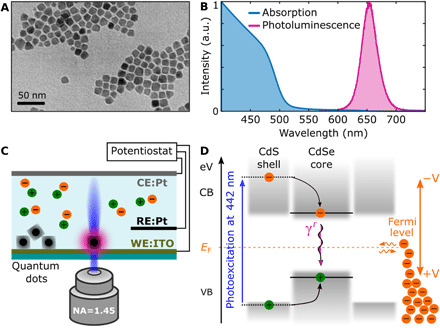
Colloidal giant-shell CdSe/CdS quantum dots and experimental setup. (**A**) TEM image of batch 2. (**B**) Absorption and photoluminescence spectra of batch 2. a.u., arbitrary units. (**C**) Sketch of a confocal microscope focused on an individual quantum dot subjected to a voltage bias between reference (RE) and working (WE) electrodes of a three-electrode electrochemical cell, while a Pt-coiled wire served as a counter electrode (CE). NA, numerical aperture. (**D**) Energy diagram of a quantum dot with valence (VB) and conduction (CB) bands accommodating an exciton. A 442-nm laser induces above bandgap excitation of the carriers in the CdS shell, which can relax to the CdSe core recombining radiatively at γ*^r^* = γ_0_ rate. The position of the Fermi level (orange dashed line) can be manipulated via the application of a voltage bias and adjusted for the electron injection into the conduction band, leading to exciton charging.

Two batches of giant-shell CdSe/CdS quantum dots were synthesized following a recently published protocol (*8*), with a minor modification (see Materials and Methods). Both batches have the same 4-nm CdSe core but with a different shell thickness, resulting in a total diameter of 10.6 ± 1.1 and 13.1 ± 2.1 nm, and we labeled them batches 1 and 2, respectively. Having two batches with different shell sizes aided us with the statistics, extension, and reliability of our findings. A representative transmission electron microscope (TEM) image for batch 2 is shown in Fig. 1A, together with the absorption and emission spectrum in Fig. 1B, showing the CdS band edge lying around 500 nm and an emission peak around 655 nm. These quantum dots exhibit nonblinking behavior at a low pump fluency (see Materials and Methods).

We excited individual quantum dots with a blue laser at 442 nm (2.8 eV) in a custom-built confocal microscope capable of recording the fluorescence with time-correlated single-photon counting, with an overall time response of 400 ps. The quantum dots were subjected to a voltage bias in an electrochemical cell, composed of a transparent indium tin oxide (ITO) working electrode and Pt quasi-reference and counter electrodes (Fig. 1C), as detailed in Materials and Methods. The position of the Fermi level (orange dashed line in Fig. 1D) was controlled by the applied voltage bias.

### Statistical scaling model for charged excitons

**Fig. 2:**
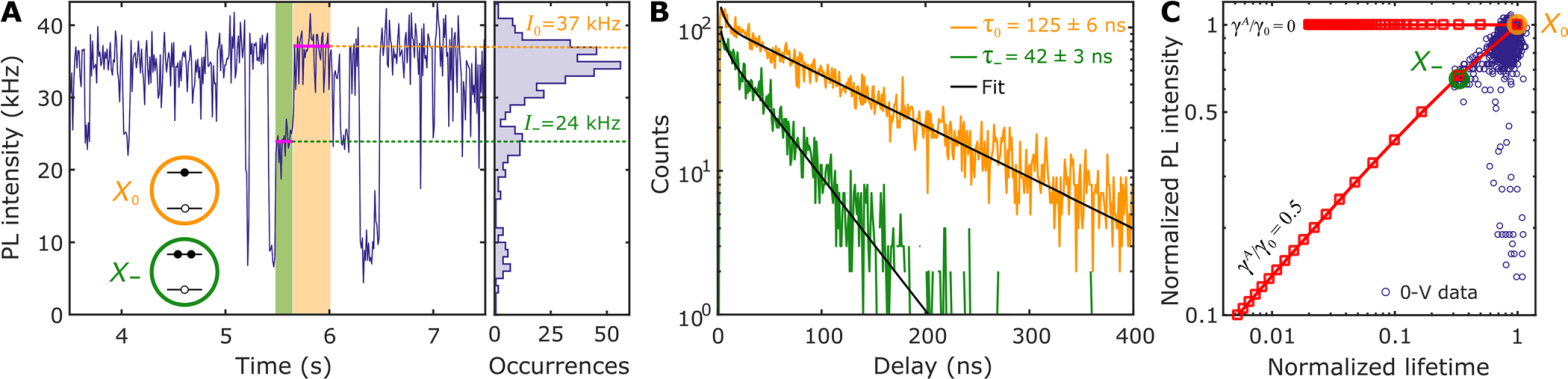
FLID at 0 V and statistical scaling model. (**A**) Photoluminescence (PL) intensity time trace of an individual quantum dot qd1 from batch 1 measured at 0-V bias and 30-nW excitation power. Neutral exciton *X*_0_ and negative trion *X*_−_ are highlighted by orange and green areas, respectively. The time bin is 10 ms. (**B**) Decay histograms of *X*_0_ and *X*_−_ were acquired using the photon arrival times from the orange and green time windows in (A). The decay histograms are fitted with a biexponential function (black lines) to filter out the contribution of positive trion and multiexciton states characterized by short lifetimes (see fig. S5). (**C**) Fluorescence lifetime intensity distribution (FLID) visualizes *X*_0_ and *X*_−_ states, with lifetime and intensity extracted from the selected green and orange time windows in (A). The blue open circles represent statistics acquired during 60 s at 0-V bias. The red squares represent the discrete statistical scaling model (with a red connecting line as a guide to the eye), according to Eqs. 1 and 2 calculated per additional electron, and its solutions are presented for the Auger-free case (γ*^A^*/γ_0_ = 0) and a quantum dot with Auger processes (γ*^A^*/γ_0_ = 0.5).

The optical properties of an individual quantum dot depend markedly on its charge state. The most common model describing the change in optical response under charging is the statistical scaling model (*15*, *31*). The model links the total decay rate (γ_*N* − 1_) and the quantum yield (QY_*N* − 1_) of excitonic states with *N* charges. In our case, because of the fast hole Auger rate in giant quantum dots, which is due to the stronger confinement (*32*), we consider only the case of an excess of electrons. In this case, the radiative recombination rate of a charged exciton formed by the coupling of *N* electrons in the conduction band and a single hole in the valence band increases with the electron number as *N*γ_0_, where γ_0_ is the radiative rate of a neutral exciton. It can be understood as an *N*-fold increase in the recombination pathways as each electron contributes. This is illustrated in Fig. 2, where *X*_0_ (orange) is the neutral exciton, and *X*_−_ (green) is the negative trion. Auger recombination is a nonradiative decay pathway, with a rate that increases with the electron number as $\gamma_{N-1}^{A}=N(N-1)\gamma^{A}$, where γ*^A^* is a constant characterizing the rate of a single electron pathway (*33*). The total recombination rate of band-edge excitons is the sum of radiative and nonradiative (Auger) rates, i.e., $\gamma_{N-1}=N\gamma_{0}+\gamma_{N-1}^{A}$. Its statistical scaling can be rewritten as (see the Supplementary Materials for a full derivation)

$$\gamma_{N-1}/\gamma_{0}=N[1+\frac{\gamma^{A}}{\gamma_{0}}(N-1)]$$ (1)

and

$$QY_{N-1}={\left[1+\frac{\gamma^{A}}{\gamma_{0}}(N-1)\right]}^{-1}$$ (2)

The Auger processes can be reduced in quantum dots with a giant shell (*30*). According to Eqs. 1 and 2, in the limit γ*^A^* ≪ γ_0_, the emission rate of charged excitons roughly scales as *N*γ_0_, and the emission intensity is similar to that of the neutral exciton *X*_0_.

A typical experimental intensity time trace collected on a quantum dot of batch 1, for 0-V applied bias, is shown in Fig. 2A (blue trace). The decay histograms (Fig. 2B) were extracted from Fig. 2A by accumulating delay times in the two time windows indicated by the orange and green shaded areas. They reveal the neutral exciton *X*_0_ and the negative charged exciton (trion) *X*_−_ as the dominant states, with single exponential decays and lifetimes of 125 ± 6 and 42 ± 3 ns, respectively. These states can be identified in a fluorescence lifetime intensity distribution (FLID), which correlates the fluorescence intensity and lifetime, as shown in Fig. 2C. The blue points in the FLID were obtained by splitting the intensity time trace in 20-ms-long time bins and computing the corresponding lifetime-intensity pair. The spread of the data points between *X*_0_ and *X*_−_ is due to the fast blinking (flickering), and thus averaging, between the two states. States observed with the same lifetime and different fluorescence intensities are an example of B-type blinking due to the hot-exciton trapping (*30*). The *X*_0_ and *X*_−_ states are located along the red line, which connects the states predicted from Eqs. 1 and 2, for γ*^A^*/γ_0_ = 0.5 ± 0.1. The latter value is extracted from the *X*_0_/*X*_−_ lifetime-intensity ratios in Fig. 2B after integrating for the orange and green time window periods. The relatively slow Auger recombination (γ*^A^*/γ_0_ = 0.5 ± 0.1) is due to the thick 3.3-nm CdS shell in batch 1, and it is even slower (γ*^A^*/γ_0_ = 0.08 ± 0.02), thus less efficient, for quantum dots in batch 2 with 35% thicker shell (4.5 nm, cfr. below).

### Control of intensity blinking

**Fig. 3:**
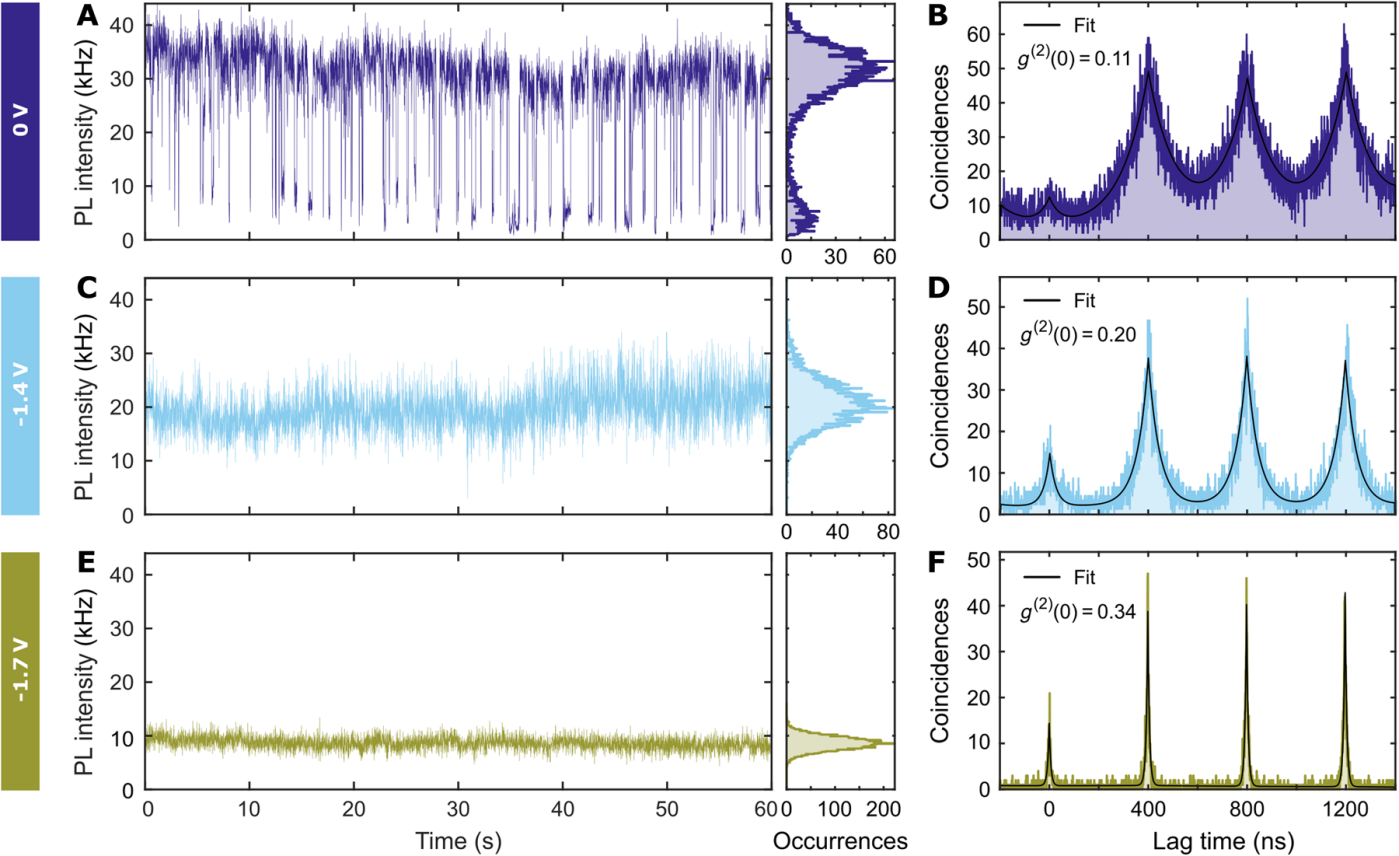
Control of blinking and intensity via application of voltage bias. (**A**, **C**, and **E**) Photoluminescence intensity time traces of the quantum dot qd1 from batch 1 obtained at 0-, −1.4-, and −1.7-V static bias. The time bin is 10 ms. (**B**, **D**, and **F**) Intensity autocorrelation *g*^(2)^ (*t*) histograms for the corresponding values of static voltage bias measured during 120 s. The measured values of *g*^(2)^ (0) confirm that the quantum dot remains a good single-photon source at negative bias. (A) At the constant bias of 0 V, we observe photoluminescence blinking between high and low emissive states, which we attribute to neutral exciton and positive trion. (C) The decrease in applied potential to −1.4 V suppresses the blinking to positive trion. (E) Further lowering of bias to −1.7 V induces formation of highly charged excitons characterized by lower emission intensity.

The intensity time trace at 0 V in Fig. 3A presents a typical blinking behavior between high (35 kHz) and low (5 kHz) emissivity states, which correspond to the neutral exciton and positive trion (see fig. S5). When the applied bias is lowered to −1.4 V (Fig. 3C), the blinking between exciton and positive trion is completely suppressed, while the blinking between negatively charged excitons still takes place, which can be seen from the wide intensity distribution in the corresponding occurrences histogram. Further lowering of the applied bias to −1.7 V induces formation of highly charged and stable excitonic states (Fig. 3E), since the applied static bias does not allow them to decay to lower charged excitonic states. Besides the reduction in blinking, the applied voltage has an effect on the quantum dot fluorescence intensity, as the average state emissivity decreases from 35 to 20 kHz for −1.4 V. This dimming progresses further when the applied bias is lowered to −1.7 V, reaching 10 kHz (Fig. 3E). By Hanbury Brown and Twiss interferometry, we verified that the second-order correlation at zero delay times *g*^(2)^(0) does not rise above 0.5 for the bias above −1.8 V, which means that the investigated quantum dot remains a single-photon emitter (Fig. 3, B, D, and F). Instead, at −1.8 V, *g*^(2)^(0) raises to 0.57 ± 0.05 (see fig. S4). The increase in the zero delay peak is assigned to an increased biexciton emission efficiency at high negative bias, as the Auger rate for holes becomes comparable to the radiative rate of charged excitons (*34*). For completeness, we report that some quantum dots displayed a photoluminescence brightening when subjected to a negative bias (see fig. S9). We attribute this to a retrieval of the exciton brightness at a negative potential similar to what has been reported in (*35*), probably due to an initial high density of defect states that were passivated.

### Control of decay rate

**Fig. 4:**
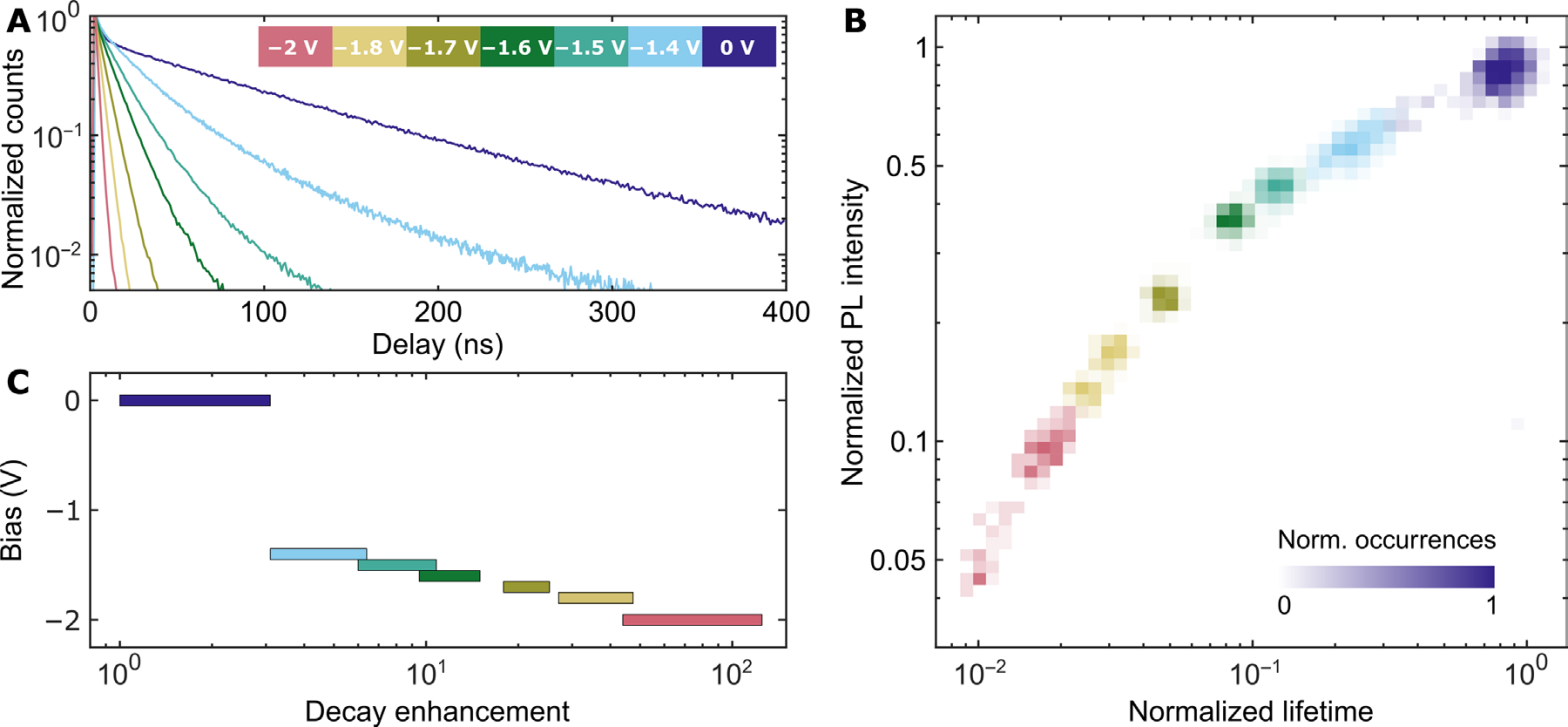
Active control of decay rate with voltage bias. (**A**) The quantum dot qd1 from batch 1 was measured at different voltage biases in the electrochemical cell (the applied voltage biases are coded with colors, as shown in the inset). The overall decay histograms acquired during 60 s demonstrate a shortening of the lifetime with increasing negative bias. (**B**) Each of the fluorescence intensity time traces, measured at static bias, was processed as described in Fig. 2, resulting in a FLID. The intensity-lifetime pairs are represented here as a normalized distribution where the number of occurrences is measured by the level of transparency (a representative scale bar is shown for 0 V). (**C**) Decay rate enhancement could be controlled by applying a voltage bias. The decay rate increased rapidly for voltages below −1.4 V. The shortest decay lifetime 0.9 ± 0.2 ns was measured at −2 V for this particular quantum dot, which corresponded to an enhancement of 140 ± 30.

The decrease in emission intensity shown in Fig. 3 is correlated with a change in decay rate. This is demonstrated in Fig. 4A for a wide range of negative voltage bias. Decay histograms integrated for 60 s for the same quantum dot as in Fig. 3 indicate a marked reduction in the fluorescence decay time, from 125 ns at 0 V down to 0.9 ns at −2 V (140-fold). The correlation between fluorescence lifetime and intensity at various negative bias can again be combined into a FLID plot, as shown in Fig. 4B. The applied bias is encoded in different color maps that represent the distribution of occurrences. The measured states monotonically decrease their lifetime and intensity upon applying of the negative bias. For each applied voltage, the quantum dot is in a well-defined lifetime-intensity state, with fluctuations mostly due to experimental noise.

### Charge-state tomography of an individual quantum dot

**Fig. 5:**
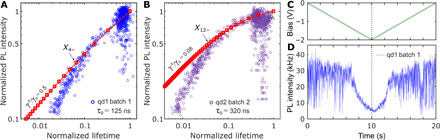
Optical response of a quantum dot during a cyclic voltammetry scan demonstrating reproducibility. (**A**) FLID of the quantum dot (qd1; blue circles) from batch 1, which has been shown in Figs. 2 to 4, extracted from intensity time traces measured during cyclic voltammetry scans, as shown in (D). (**B**) FLID of another quantum dot, from batch 2 (qd2; purple circles). In (A) and (B), the red lines represent the statistical scaling model for different Auger rates according to Eqs. 1 and 2. In the cyclic voltammetry scans, the bias was varied linearly in time, as indicated in (**C**). At *t* = 10 s, the scan polarity was reversed to return to the initial bias of 0 V. Positive bias caused quenching of the neutral exciton (see an example in fig. S6). (**D**) Intensity time trace measured during the potential scan of qd1 from batch 1 used to build FLID in (A). After the suppression of blinking around −1.4 V, the photoluminescence intensity was gradually quenched under the linear decrease in the applied potential. The initial photoluminescence intensity was restored when the scan polarity was reversed (an intensity trace of this quantum dot during six voltammetric cycles is shown in fig. S8) and began to blink again around −1.4 V. The time bin is 10 ms.

Figure 5 (A and B) plots the FLID data for both batches, when the bias was continuously varied in a voltage scan from 0 to −2 V, as shown in Fig. 5C. Cyclic voltammetry scan allowed us to span the charge-state configuration space in a short time (20 s), minimizing optical misalignment issues. Obtained results were similar to what is observed at static bias in Fig. 4.

The red line in Fig. 5 (A and B) represents the statistical scaling model according to Eqs. 1 and 2, where the relative Auger rates γ*^A^*/γ_0_ were obtained, for batch 1, from the lifetime-intensity ratios of exciton and trion, as described in the “Statistical scaling model for charged excitons” section. Instead, for batch 2, we extracted γ*^A^*/γ_0_ from the fit of the lifetime-intensity distribution of the neutral and low charged excitons, i.e., beyond the trion, as the quantum yields of exciton and trion are too similar and the Auger rate estimation has a very large error (see fig. S12). The evolution of intensity and lifetime for low bias, up to *X*_4−_ for batch 1 and *X*_12−_ for batch 2, can be fitted with the statistical scaling model, which is, in essence, a single-particle model that assumes a fixed electron-hole overlap and thus neglects many-body Coulomb interactions (*22*). For lower negative bias, the quantum dots in batches 1 and 2 are charged beyond *X*_4−_ and *X*_12−_ states, respectively, and a pronounced deviation is visible in Fig. 5 (A and B) (see Materials and Methods). In this case, the FLID cannot be fitted with Eqs. 1 and 2. Considering that previous results on charged excitons and biexcitons already confirmed that an ad hoc β factor should be used to improve the agreement between lifetime and fluorescence intensity in the scaling model (*22*, *31*), we postulate that the higher carrier densities created here will only lead to a further modification of the electron wave function, affecting both electron-hole overlap (and thus γ_0_) and the Auger recombination rate γ*^A^*, which is highly sensitive to the behavior of the electron wave function at the CdSe/CdS interface (*36*). The lowest applied potential of −2 V causes a large decrease in the lifetime, with largest recorded values of 140 ± 30–fold, while the intensity drops by a factor of 25 ± 4 for batch 1 and 210 ± 40 and 12 ± 3 for batch 2, respectively. These values of decay enhancement and intensity drop were limited by the lowest voltage bias −2 V, which did not cause the degradation of ITO substrate.

## DISCUSSION

Coulomb repulsion upon charging, especially in air or vacuum, can limit the charge state attainable. However, in our experiments in liquid, adsorption of tetrabutylammonium (TBA) cations compensates the electrochemical buildup surface potential due to the injected charges (*37*). These screened charges reduce the overpotential requirements for further charge injection (*28*). In a simplified estimation for our giant-shell quantum dots, each quantum dot (diameter, ∼12 nm) can allocate up to ∼100 TBA cations (radius, ∼0.5 nm) on its surface. Therefore, we conclude that Coulomb repulsion is not the dominant effect due to the larger surface area in our quantum dots.

Once the bias is increased to reach the band edge, electron injection depends on the available states. From a density of states reasoning, we have calculated the expected level spacing (details in the Supplementary Materials), and we confirm theoretically that for a voltage of 80 to 150 meV above the conduction band edge, ∼20 states can be populated. Switching of the photodynamics from a charging state to another can be performed by simply applying the voltage, with a rise time of about 2 μs, limited by our electronics (see fig. S8).

Last, we discuss the repeatability and reproducibility of the results. We repeated this experiment with 37 individual quantum dots from two batches, and we observed charging beyond doubly negative charged exciton in 13 cases, which is around 30%, while in the other cases, the quantum dots did not demonstrate the lifetime-intensity dip as in Fig. 5D (see fig. S7 and table S1). Moreover, it has been recently pointed out that charging-induced damage can occur because of reduction in the quantum dot surface (*38*). Here, the application of up to −2 V of negative potential is reversible and does not damage the quantum dot (Fig. 5D). We believe that this is because the quantum dot thick shell can accommodate many defect states. In our experiments, the voltage bias was gradually varied, and Fig. 5D shows a clear drop in the emitted intensity for voltages below −1.4 V, which recovers when the bias is returned to 0 V. This recovery can be repeated many times with no sign of degradation in the optical properties (fig. S8): We tested it up to 540 cycles during 3 hours, a time span limited by the degradation of the ITO working electrode. Besides, here, the heating of quantum dots is not a concern, as we are not inducing photon absorption as in photoexcitation experiments.

Controlling the charge state of an individual quantum dot can be very important for quantum technologies, where the undesired switching to a different charge state precludes interfacing the electronic spin to photons (*39*). Boosting the decay rate now brings the colloidal nanocrystals on par with the fluorescence lifetime of nitrogen vacancy (NV) centers in diamond (*40*), as well as epitaxial quantum dots (*41*), and could open a path toward coherent emission at room temperature once the decay rate becomes faster than the decoherence rate (*42*). An intensity-switchable nanoscale light source can find important applications for optical signal processing with very stable giant quantum dots, where the switching speed is usually limited by the decay rate of the quantum dot, which can here instead reach gigahertz speeds when the exciton is maximally charged.

In conclusion, we report the observation of highly charged excitons, which induces a greater than 210-fold increase in the decay rate, with only a 12-fold reduction in the quantum yield. The charging process is reversible and deterministic, allowing for direct manipulation of the quantum dot emission rate through the applied bias, while preserving the single-photon emission characteristics. The fluorescence intensity-lifetime relation observed at high charge density goes beyond the conventional statistical scaling model, and not all quantum dots show the lifetime reduction, indicating the need for a more general model, including many-body corrections. Charging colloidal quantum dots is a powerful route for enhancing and controlling their photodynamics, which has important implications for tunable quantum sources, brighter displays, and optical signal processing and can lead to previously unknown approaches to charge/voltage sensing.

## MATERIALS AND METHODS

### Quantum dots

CdSe/CdS core/shell quantum dots were synthesized using established methods (*8*), with a small modification: Before CdS shell growth, CdSe core quantum dots were suspended in ODE (octadecene), together with 0.25 ml of a 0.5 M solution of cadmium oleate in ODE. This mixture was degassed for 30 min at 110°C and subsequently heated to 300°C. Next, an equimolar mixture a 0.5 M cadmium oleate solution in ODE, and a 0.5 M TOPS (trioctylphosphine sulfide) solution was then slowly injected (at a rate of about 1 ml/hour) by a syringe pump to grow the CdS shell, with the total amount of Cd and S precursors determined by the desired shell thickness. Individual quantum dots from both batches exhibited nonblinking photodynamics at a low pump fluency and blinking at a high pump fluency, as in the presented here experiments (see fig. S2).

We remark here that we used a very short time bin (10 ms) to plot the intensity time traces, much shorter than in most reported nonblinking quantum dots experiments (30 to 200 ms) (*22*, *30*, *43*, *44*), which is also why we can capture very fast-blinking events.

### Electrochemistry

The experimental setup consisted of a custom-built three-electrode electrochemical cell mounted on a time-resolved confocal fluorescence microscope. Diluted quantum dots in toluene were spin-coated at ITO substrates (70 to 100 ohms per square; Diamond Coatings), which was the working electrode of the electrochemical cell. Coiled and straight Pt wires served as counter and quasi-reference electrodes, respectively. The distance between working and counter electrodes was 0.5 cm. The electrolyte was 0.1 M TBA hexafluorophosphate (≥99.0%; Sigma-Aldrich) in acetonitrile (99.8%; Sigma-Aldrich) or propylene carbonate (99%; Sigma-Aldrich). The voltage bias between the reference and working electrodes was controlled with a CHI 760C potentiostat (CH Instruments). As an almost negligible ohmic drop was determined for our setup (up to 9 mV, cf. notes in the Supplementary Materials), all voltage biases are reported, as recorded, i.e., without *iR* drop correction and versus the Pt quasi-reference electrode. The Pt quasi-reference electrode potential against Ag/AgCl(sat) was measured to be 57 ± 2 mV under our experimental conditions [cf. notes in the Supplementary Materials for conversion of the potential values into normal hydrogen electrode scale].

### Lifetime measurements

We used a blue laser (LDH-D-C-440, PicoQuant) at 442 nm with a pulse width of 64 ps and a repetition frequency of 2.5 MHz to excite quantum dots. Samples were scanned using a three-dimensional piezo stage (E-545.3CD PI Nano). A high–numerical aperture (NA) oil-immersion objective (Plan Apochromat 100×; NA, 1.45) focused the laser beam on an individual quantum dot and collected the photoluminescence signal using an avalanche photo diode (SPCM-AQRH, PerkinElmer) connected to a time-correlated single-photon counting module (TimeHarp 260, PicoQuant). Photoluminescence decay histograms were obtained by recording the time between a laser excitation pulse and arrival time of a photon at a detector.

### Photon antibunching

The collected photoluminescence signal from a quantum dot was tested in a Hanbury Brown and Twiss interferometer to verify the single-photon emission nature. The setup consisted of a 50/50 beam splitter and two avalanche photo diodes, which detected the arrival times of photons to build a coincidence histogram. The second-order correlation function *g*^(2)^(0) was measured by comparing the peak area at zero arrival time with the area averaged over the first three lagging peaks without any background subtraction.

### Charge state of exciton

We extract the excitonic charge state by fitting the intensity-lifetime evolution with the statistical scaling model, which is valid for low charge states, assuming a fixed electron-hole overlap and neglecting many-body effects. The fit is robust; we have tested it by varying the initial conditions and the fitting range, and we got a variability in the range of 4 to 6 electrons for batch 1 and 12 to 16 electrons for batch 2 (see fig. S12). We report a conservative estimation of the exciton charging (4 and 12 for batches 1 and 2, respectively), which was extracted from the point where the statistical scaling model starts to deviate strongly from the experimental results, which is *X*_4−_ for batch 1 and *X*_12−_ for batch 2 (see Fig. 5, A and B).

## Supplementary Material

abb1821_SM.pdf

## SUPPLEMENTARY MATERIALS

Supplementary material for this article is available at http://advances.sciencemag.org/cgi/content/full/6/38/eabb1821/DC1
