# Supplementary material for: Electrical control of single-photon emission in highly charged individual colloidal quantum dots
Source: Sci Adv. 2020 Sep 18;6(38):eabb1821. doi: 10.1126/sciadv.abb1821 (PMC7500932; doi:10.1126/sciadv.abb1821)
Supplement: abb1821_SM.pdf [file abb1821_SM.pdf]

Science Advances
AAAS

advances.sciencemag.org/cgi/content/full/6/38/eabb1821/DC1

Supplementary Materials for

Electrical control of single-photon emission in highly charged individual colloidal quantum dots

Sergii Morozov, Evangelina L. Pensa, Ali Hossain Khan, Anatolii Polovitsyn, Emiliano Cortés, Stefan A. Maier, Stefano Vezzoli, Iwan Moreels, Riccardo Sapienza*

*Corresponding author. Email: r.sapienza@imperial.ac.uk

Published 18 September 2020, *Sci. Adv.* **6**, eabb1821 (2020)
DOI: 10.1126/sciadv.abb1821

This PDF file includes:

Quantitative determination of the degree of charging.
Blinking dynamics.
FLID modeling: The statistical scaling model.
Ohmic drop.
Conversion of the electrochemical potential scale into the normal hydrogen electrode potential one.
Electrochemical stability of quantum dots.
Figs. S1 to S12
Table S1
References

Quantitative determination of the degree of charging. Considering a CdS band gap of ca. 2.4 eV and a Fermi level centred near the middle of the gap, we estimate that we need to raise the bias to 1.2 V before we start to inject electrons in the conduction band, in line with the observed threshold of 1.4 V. In addition, we can estimate the bias needed to inject N electrons, by considering that the large CdS shell allows us to approximate the band structure by bulk CdS ($\sqrt{E}$ dependence). The calculation yields following bias V_{fin} (referenced to $V = 0$ eV at the CdS conduction band edge) to fill the first N states with electrons:

$$V_{fin} = (\pi \cdot N)^{2/3} \cdot 2 \cdot \hbar^2/(m_e \cdot d^2) \cdot 1/e. \tag{1}$$

Here, m_e is the reduced CdS electron mass for (0.16 m_0, m_0 is the free electron mass), d is the quantum dot diameter, and the final term $1/e$ is inserted to obtain V_{fin} on an eV scale.

Taking $N = 20$, and $d = 10 - 15$ nm, we find $V_{fin} = 80 - 150$ meV, well in line with what we observe experimentally.

Blinking dynamics. We demonstrate electric control over the photodynamics of individual quantum dots, and focus our investigation to high-quality quantum dots, which are both the most common dots used at present, see for example [44], and which are truly single photon source. Moreover, the blinking dynamics strongly depend on the excitation power, or pump fluency and our quantum dots exhibited almost non-blinking behaviour at a low pump fluency ($\langle\eta\rangle = 0.01$), however exhibited blinking when the pump fluency was increased as it is demonstrated in Fig.S2. The results presented in the main text was obtained at the high pump fluency corresponding to 20 nW of excitation power to demonstrate the control on blinking dynamics.

FLID modelling: the statistical scaling model. Statistical scaling of the radiative rate of a charged state with N electrons and 1 hole is:

$$\gamma^r_{N-1} = N\gamma_0, \tag{2}$$

where γ_0 is a radiative rate of the neutral exciton, while the nonradiative Auger rate for electrons scales as

$$\gamma^A_{N-1} = N(N-1)\gamma^A \tag{3}$$

(see [15]).

We assume here that the Auger recombination rate of holes is much larger than that of electrons.

The total recombination rate:

$$\gamma_{N-1} \equiv \gamma^r_{N-1} + \gamma^A_{N-1}. \tag{4}$$

Thus, the normalised lifetime of a charged state with N electrons to that of neutral exciton:

$$\frac{\tau_{N-1}}{\tau_0} = \frac{\gamma_0}{\gamma_{N-1}} = \frac{1}{N} \cdot \frac{1}{1 + \tau_0(N-1)\gamma^A}, \tag{5}$$

where $\tau_0 = 1/\gamma_0$ is the lifetime of neutral exciton.

Measured intensities on the experiment are proportional to the quantum yield, therefore the normalised intensity of a charged state with N electrons (assuming the neutral exciton QY_0 is 1):

$$\frac{I_{N-1}}{I_0} = \frac{QY_{N-1}}{QY_0} = \frac{\gamma^r_{N-1}}{\gamma_{N-1}} = N\frac{\tau_{N-1}}{\tau_0}. \tag{6}$$

Ohmic drop. The Ohmic drop was determined by analysing the Bode plot obtained by impedance spectroscopy. Electrochemical impedance spectra were recorded at 0V with an amplitude of 5 mV, in the frequency range between 10^5 to 0.1 Hz. The average resistance for our home-built cell was determined to be R = $89 \pm 4\ \Omega$ giving a negligible Ohmic drop of ca. 9 mV.

Conversion of the electrochemical potential scale into the normal Hydrogen electrode potential (NHE) one. Open circuit potential measurements of the Pt quasi reference electrode (Pt_{QRE}) against the Ag/AgCl(sat) electrode were performed in the same conditions as the quantum dot electro-charging experiments. We found that under those experimental conditions the potential of Pt_{QRE} was 57 ± 2 mV vs. Ag/AgCl(sat). Hence, since the potential of the Ag/AgCl(sat) vs. NHE is 199 mV (see Section 5.2 in [45]), the potential of the Pt_{QRE} vs NHE is 256 mV and the interconversion of scales can be accomplished accordingly to E(vs NHE) = E(vs Pt_{QRE}) - 0.256 V.

Electrochemical stability of quantum dots. Fig. S8 a,b shows that the photoluminescence response is reversible along cycles independently on how the potential is applied between 0 V and -2 V (i.e. linear or stepwise). The intensity is decreased/recovered at about the same potential, without significant changes in the average intensity for each state.

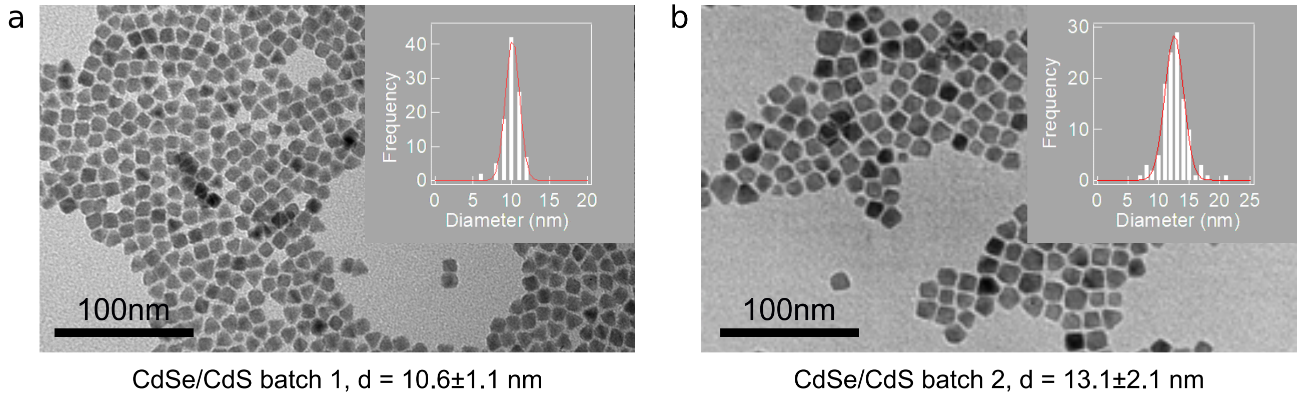

FIG. S1. **Size distribution of CdSe/CdS quantum dots in batch 1 and 2.** TEM images and quantum dot size distribution histograms of **a** batch 1, and **b** batch 2. A 4 nm CdSe core was used in the synthesis of both batches.

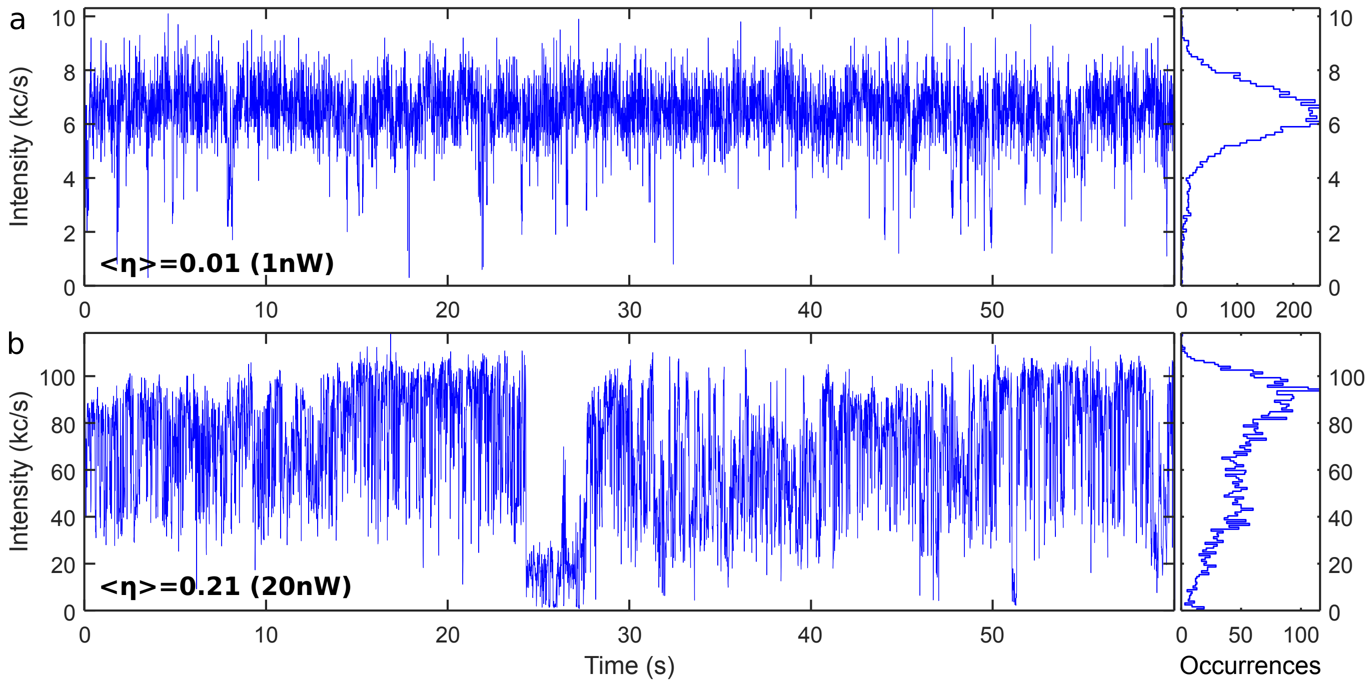

FIG. S2. **Pump fluency $\langle\eta\rangle$ affects blinking dynamics of an individual quantum dot.** A giant shell quantum dot from batch 1 exhibits almost non-blanking photoluminescence dynamics at low pump fluency $\langle\eta\rangle = 0.01$ as shown in panel **a**, however the increase of pump fluency to $\langle\eta\rangle = 0.21$ results in a complex blinking dynamics as shown in panel **b**. The time bin of intensity time traces is 10 ms. The panels to the right of intensity time traces demonstrate intensity occurrence histograms, which present the distribution of photoluminescence intensity measured during the experiment.

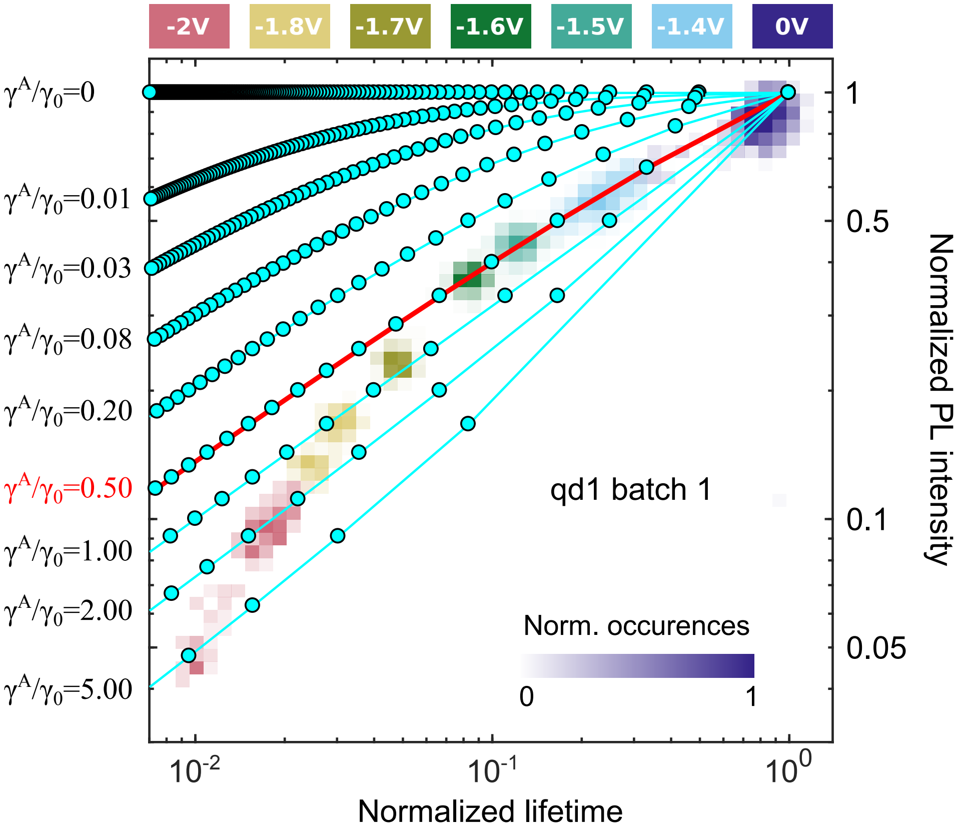

FIG. S3. **The scaling model does not follow the experimental charging route.** The statistical scaling model describes the intensity-lifetime correlation of the lowest negatively charged excitons as indicated in the FLID from Fig.4b by red curve obtained for $\gamma^A/\gamma_0 = 0.5$. The statistical scaling model with the only one free parameter γ^A cannot fit the charging route in the FLID as shown for the relative Auger rates in range $\gamma^A/\gamma_0 = 0 \div 5$.

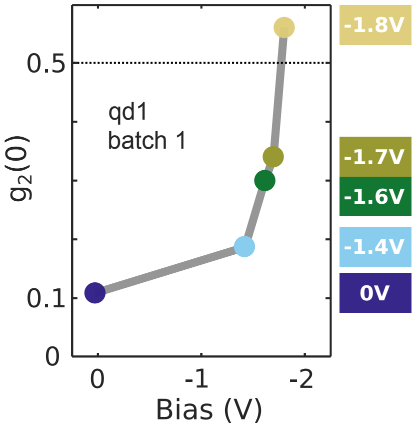

FIG. S4. **Antibunching vs applied bias for *qd1* from batch 1 presented in the main text.**

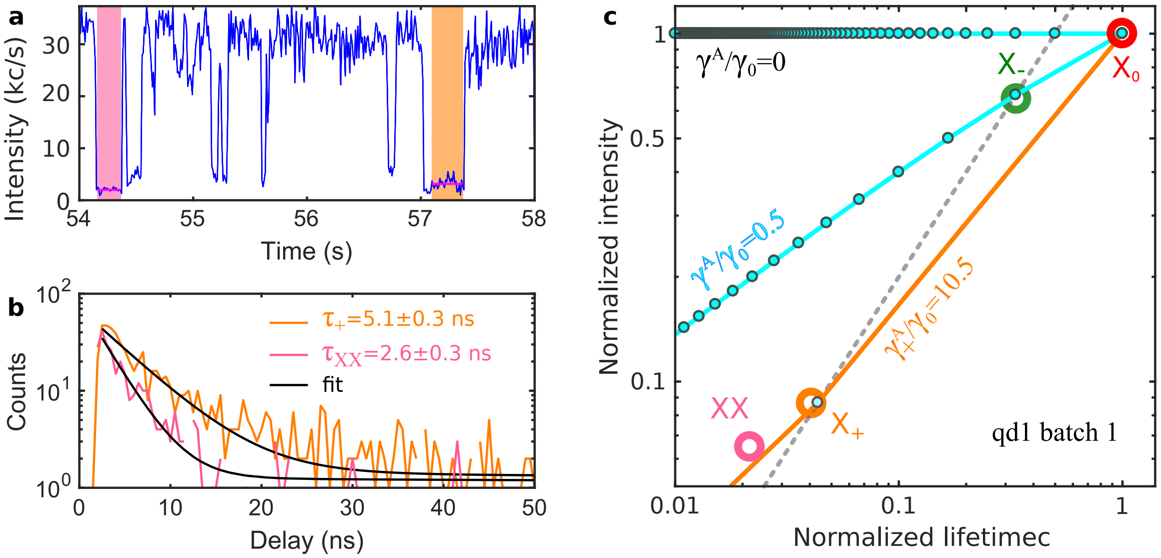

FIG. S5. **Characterisation of the low emissivity states in intensity time trace at 0 V. a** Intensity time trace of a quantum dot *qd1* from batch 1 was measured applying 0 V bias, and it reveals blinking between high and low emissivity states. **b** The decay histograms were acquired from the highlighted bins in **a**, and are characterised by different short lifetimes. **c** FLID summarises extracted excitonic states. The high emissivity state has a lifetime of 125 ± 6 ns and it is assigned to the neutral exciton, while the low emissivity state in the orange time window has a shorter lifetime 5 ± 0.3 ns and assigned to the positive trion. This state assignment was confirmed by the excellent agreement between the experimentally measured biexciton lifetime $\tau_{XX}^{exp} = 2.6 \pm 0.3$ ns (pink time window) and the one predicted by the statistical scaling model $\tau_{XX}^{calc} = 2.4$ ns (for details see [22]). The grey dashed line goes through both negatively and positively charged excitons, which also confirms that they both are trions. The Auger rate for positive trion is about 21 times faster then the one for negative trion.

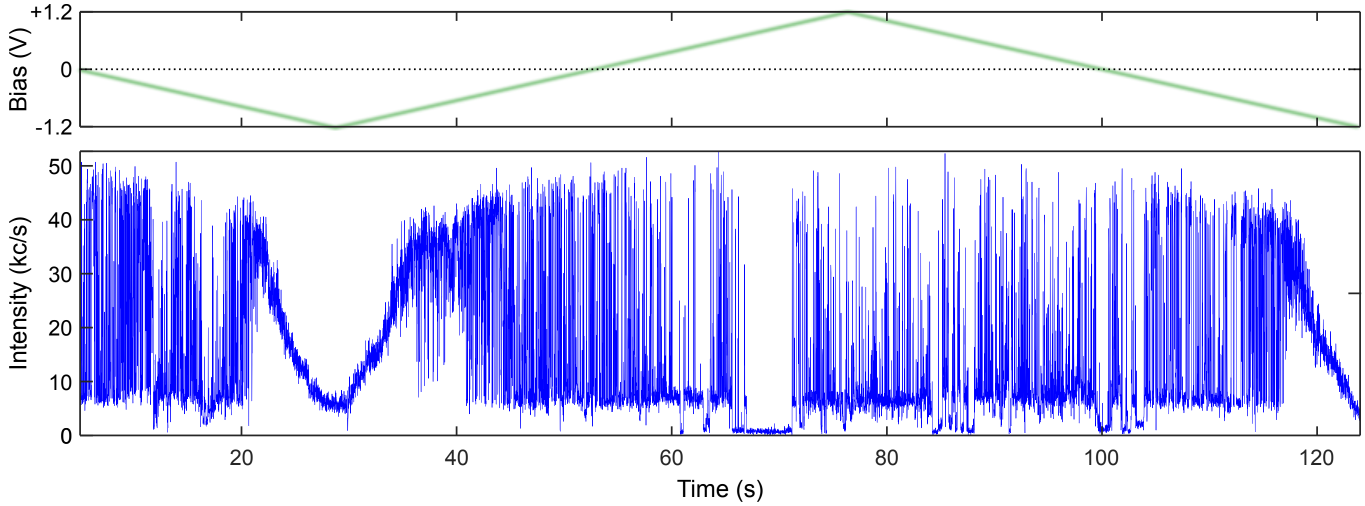

FIG. S6. **Positive voltage bias does not control emission dynamics of a single quantum dot.** Cyclic voltammetry scan in range ±1.2 V is shown for the quantum dot qd2 from batch 2, which is reported in Fig.5b. The positive bias was applied between 50 s and 100 s of the intensity time trace.

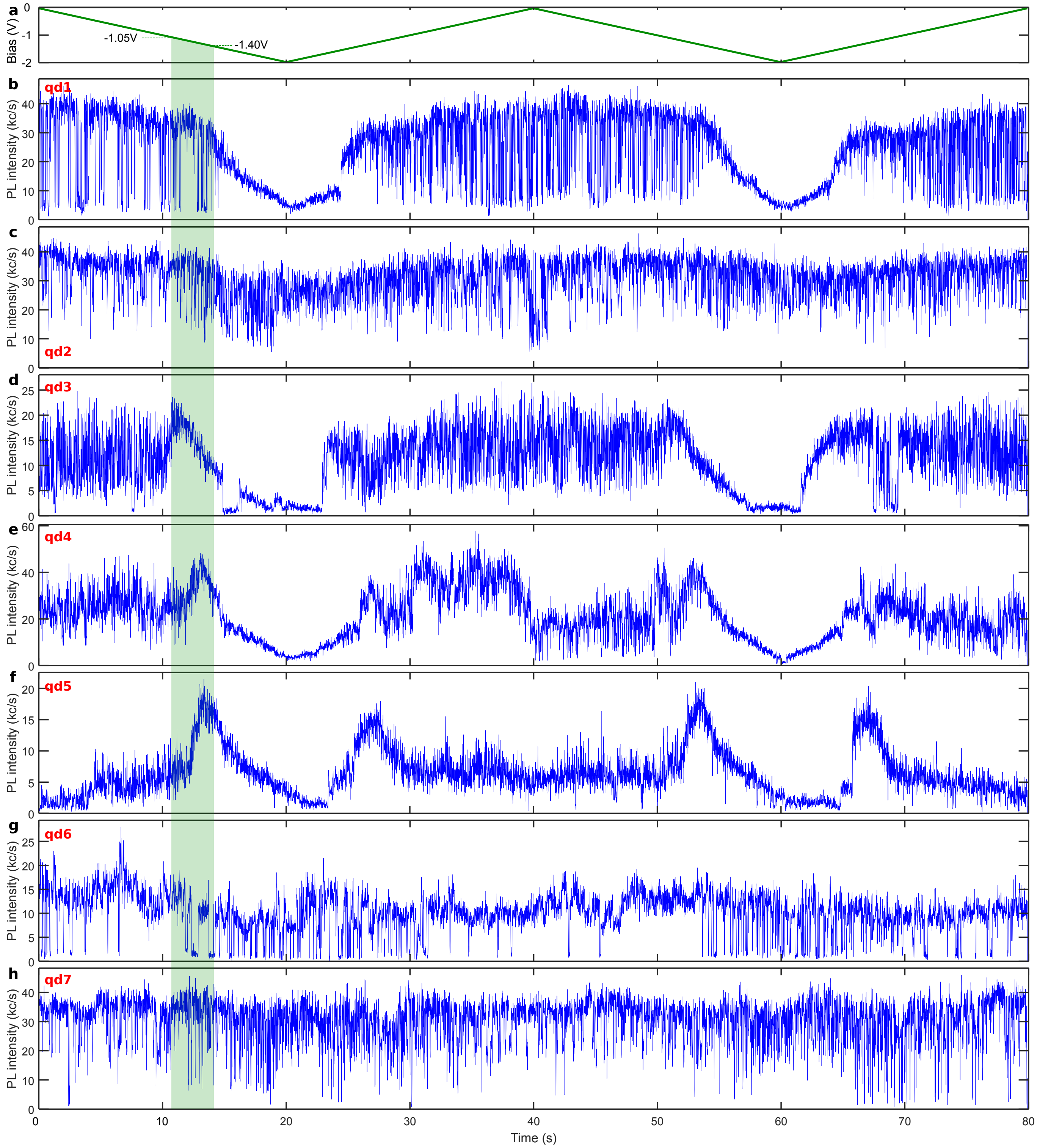

FIG. S7. **Statistics of response of quantum dots in batch 1 to negative voltage.** Photoluminescence intensity profiles in **b-h** of 7 individual quantum dots within the same electrochemical cell were measured during cycling voltammetry scans from 0 to -2 V as shown in panel **a**. Photodynamics of quantum dots in panels **c,g,h** was not affected by the voltage bias of -2 V. The quantum dots in panels **b,d,e,f** exhibited the intensity-lifetime dip at negative bias in range from -1.05 V to -1.4 V as indicated by green shaded area.

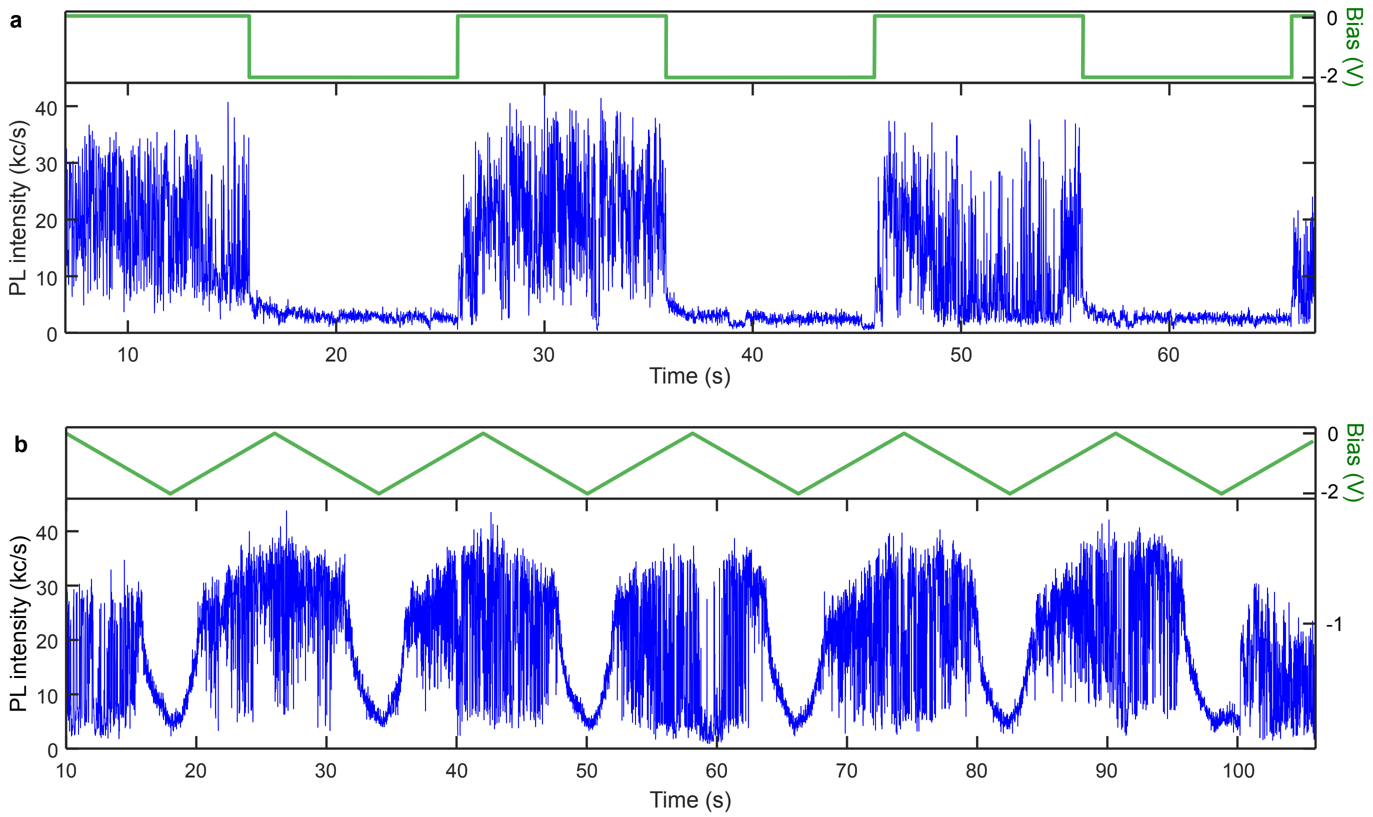

FIG. S8. **Reproducibility of quantum dot response at negative potentials.** **a** Intensity time trace of the quantum dot qd1 from batch 1 was measured when applying stepwise potential as shown with green voltage trace. The photoluminescence intensity trace demonstrates almost immediate response of the quantum dot to -2 V as well as 0 V potentials. **b** Photoluminescence intensity profile was measured during cycling voltammetry scan as in Fig.5d in the main text.

	**Lowest applied voltage bias (V)**	τ_0 **(ns)**	τ_N **at the lowest potential (ns)**	**decay rate enhancement**
$qd1$ **batch 1**	-2	125 ± 6	0.9 ± 0.2	139 ± 32
$qd2$ batch 1	-2	72 ± 5	0.8 ± 0.2	90 ± 23
$qd3$ batch 1	-2	112 ± 6	1.2 ± 0.2	93 ± 16
$qd4$ batch 1	-2	78 ± 5	0.9 ± 0.2	87 ± 20
$qd5-7$ batch 1	-2	na	na	(*)
$qd1$ batch 2	-2	415 ± 36	4.2 ± 0.3	99 ± 11
$qd2$ **batch 2**	-1.5	320 ± 25	1.5 ± 0.2	213 ± 33
$qd3$ batch 2	-2	256 ± 19	2.4 ± 0.2	107 ± 12
$qd4-30$ batch 2	-2	na	na	(*)

TABLE S1. **Statistics of quantum dot charging in an electrochemical cell.** (*) These quantum dots exhibited charging up to doubly or triply negatively charged excitons, and their intensity time traces did not have pronounced intensity dips (see for example intensity traces in Fig.S7c,h). The effect could be observed at lower applied bias, however it was causing the cell to degrade quickly and no data could be acquired. The quantum dots in bold correspond to ones presented in Figure 5 of the main text.

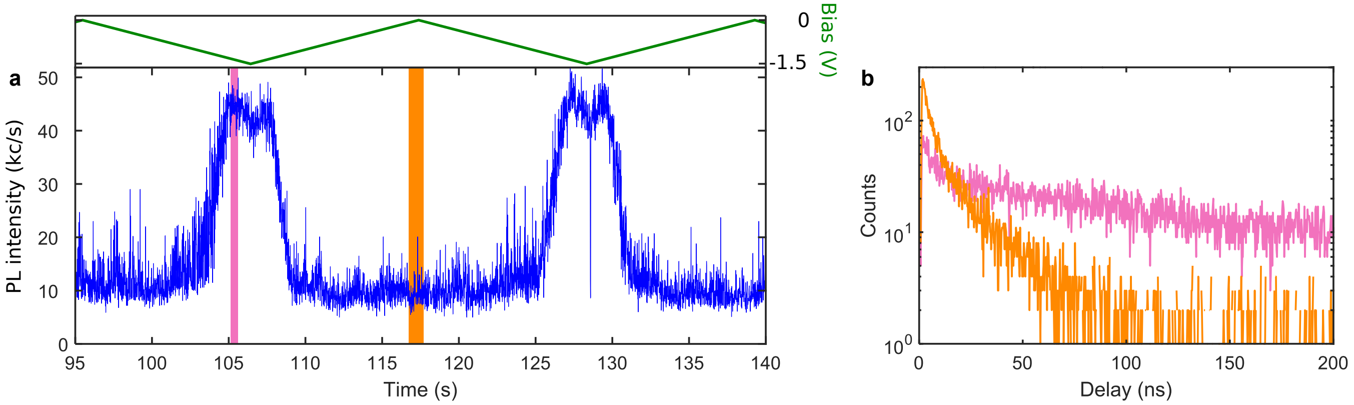

FIG. S9. **Photoluminescence of a quantum dot in a dim parasitic state can be restored by applying a negative potential.** **a** Intensity time trace of a quantum dot from batch 2 was measured during cyclic voltammetry scan (green trace in the top panel). The photoluminescence intensity rapidly increases at negative potential of -1.3 V, and decreases back to the low emissivity level when the voltage scan is reversed. **b** Decay histograms acquired during the colour windows highlighted in **a** demonstrate that the emission lifetime at negative potential of -1.3 V (pink) is longer than that at 0 V (orange). This is attributed to the trap filling at negative potential and blocking of a competing non-radiative decay channel. See another examples for quantum dots from batch 1 in Fig.S7e,f.

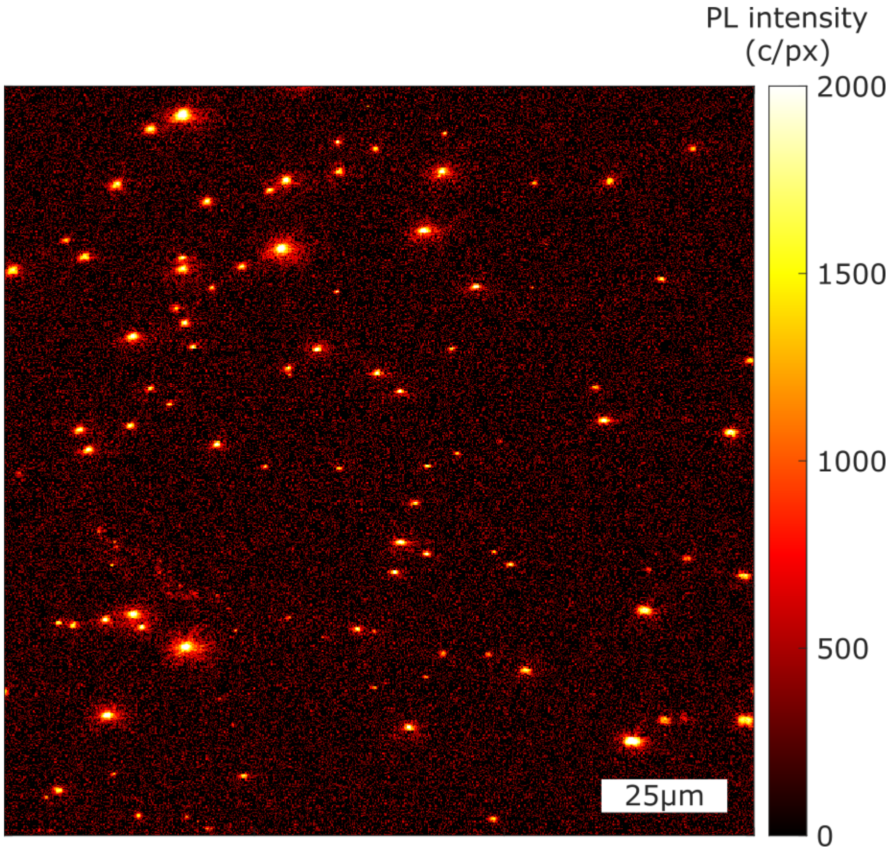

FIG. S10. **Photoluminescence confocal scan of quantum dots from batch 2 in the electrochemical cell filled with an electrolyte at 0 V bias.** 150x150 μm 2D scan of spincoated quantum dots from μM solution in toluene on an ITO substrate. The dimmest spots relate to individual quantum dots, as confirmed by the antibunching measurements.

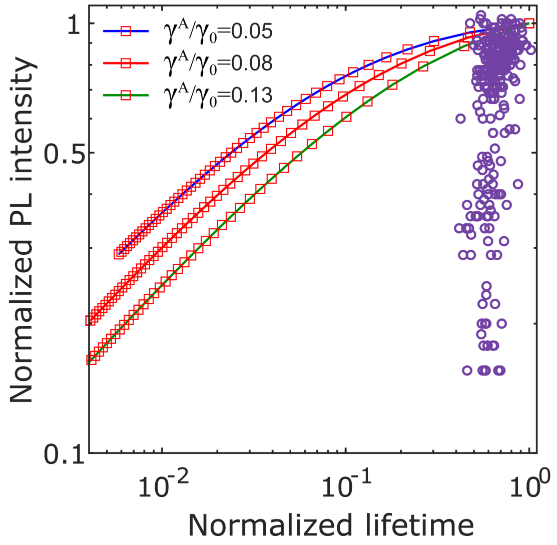

FIG. S11. **FLID of qd2 from batch 2 at 0V bias.** The intensity of negatively charged exciton is similar to the one of the neutral exciton.

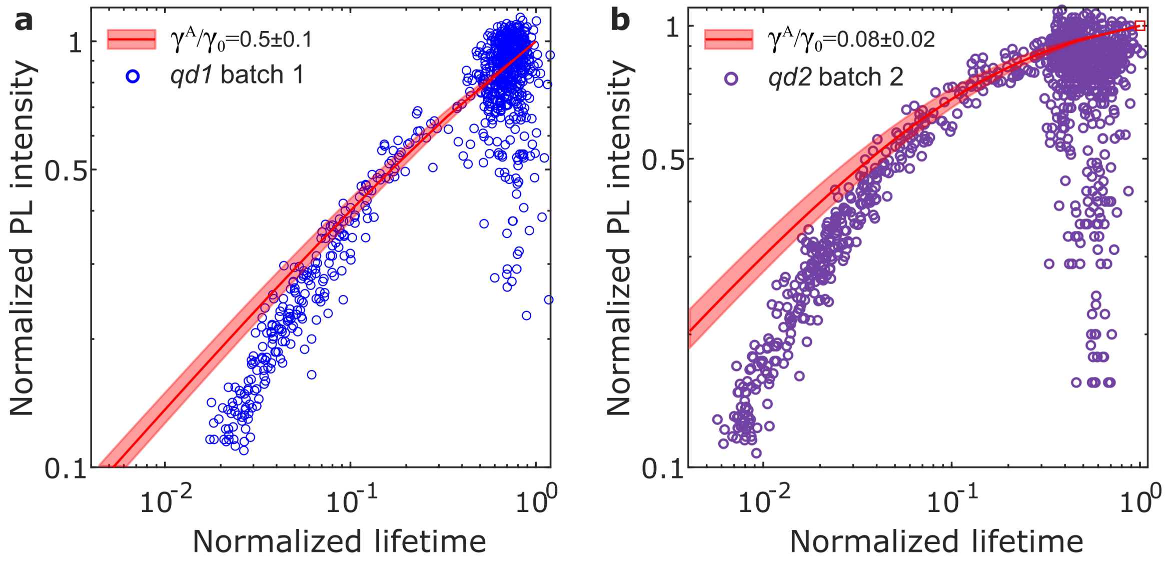

FIG. S12. **Estimation of error bounds for the relative Auger recombination.** The shaded red area represents the error for **a** qd1 batch 1, and **b** qd2 batch 2 (see inset).

REFERENCES AND NOTES

1. J. S. Steckel, J. Ho, C. Hamilton, J. Xi, C. Breen, W. Liu, P. Allen, S. Coe-Sullivan, Quantum dots: The ultimate down-conversion material for LCD displays. *J. Soc. Inf. Disp.* **23**, 294–305 (2015).

2. J. Lim, Y.-S. Park, K. Wu, H. J. Yun, V. I. Klimov, Droop-free colloidal quantum dot light-emitting diodes. *Nano Lett.* **18**, 6645–6653 (2018).

3. O. V. Kozlov, Y.-S. Park, J. Roh, I. Fedin, T. Nakotte, V. I. Klimov, Sub–single-exciton lasing using charged quantum dots coupled to a distributed feedback cavity. *Science* **365**, 672–675 (2019).

4. X. Lin, X. Dai, C. Pu, Y. Deng, Y. Niu, L. Tong, W. Fang, Y. Jin, X. Peng, Electrically-driven single-photon sources based on colloidal quantum dots with near-optimal antibunching at room temperature. *Nat. Commun.* **8**, 1132 (2017).

5. F. Pisanello, G. Leménager, L. Martiradonna, L. Carbone, S. Vezzoli, P. Desfonds, P. D. Cozzoli, J.-P. Hermier, E. Giacobino, R. Cingolani, M. De Vittorio, A. Bramati, Non-blinking single-photon generation with anisotropic colloidal nanocrystals: Towards room-temperature, efficient, colloidal quantum sources. *Adv. Mater.* **25**, 1974–1980 (2013).

6. W. K. Bae, L. A. Padilha, Y.-S. Park, H. McDaniel, I. Robel, J. M. Pietryga, V. I. Klimov, Controlled alloying of the core–shell interface in CdSe/CdS quantum dots for suppression of Auger recombination. *ACS Nano* **7**, 3411–3419 (2013).

7. B. Ji, E. Giovanelli, B. Habert, P. Spinicelli, M. Nasilowski, X. Xu, N. Lequeux, J.-P. Hugonin, F. Marquier, J.-J. Greffet, B. Dubertret, Non-blinking quantum dot with a plasmonic nanoshell resonator. *Nat. Nanotechnol.* **10**, 170–175 (2015).

8. S. Christodoulou, G. Vaccaro, V. Pinchetti, F. De Donato, J. Q. Grim, A. Casu, A. Genovese, G. Vicidomini, A. Diaspro, S. Brovelli, L. Manna, I. Moreels, Synthesis of highly luminescent wurtzite CdSe/CdS giant-shell nanocrystals using a fast continuous injection route. *J. Mater. Chem. C* **2**, 3439–3447 (2014).

9. A. F. Koenderink, Single-photon nanoantennas. *ACS Photonics* **4**, 710–722 (2017).

10. F. Nicoli, T. Zhang, K. Hübner, B. Jin, F. Selbach, G. Acuna, C. Argyropoulos, T. Liedl, M. Pilo-Pais, DNA-mediated self-assembly of plasmonic antennas with a single quantum dot in the hot spot. *Small* **15**, 1804418 (2019).

11. S. Mignuzzi, S. Vezzoli, S. A. R. Horsley, W. L. Barnes, S. A. Maier, R. Sapienza, Nanoscale design of the local density of optical states. *Nano Lett.* **19**, 1613–1617 (2019).

12. C. T. Yuan, Y. C. Wang, H. W. Cheng, H. S. Wang, M. Y. Kuo, M. H. Shih, J. Tang, Modification of fluorescence properties in single colloidal quantum dots by coupling to plasmonic gap modes. *J. Phys. Chem. C* **117**, 12762–12768 (2013).

13. C. Belacel, B. Habert, F. Bigourdan, F. Marquier, J.-P. Hugonin, S. Michaelis de Vasconcellos, X. Lafosse, L. Coolen, C. Schwob, C. Javaux, B. Dubertret, J.-J. Greffet, P. Senellart, A. Maitre, Controlling spontaneous emission with plasmonic optical patch antennas. *Nano Lett.* **13**, 1516–1521 (2013).

14. S. Morozov, M. Gaio, S. A. Maier, R. Sapienza, Metal–dielectric parabolic antenna for directing single photons. *Nano Lett.* **18**, 3060–3065 (2018).

15. C. Galland, Y. Ghosh, A. Steinbrück, M. Sykora, J. A. Hollingsworth, V. I. Klimov, H. Htoon, Two types of luminescence blinking revealed by spectroelectrochemistry of single quantum dots. *Nature* **479**, 203–207 (2011).

16. Z. Ding, B. M. Quinn, S. K. Haram, L. E. Pell, B. A. Korgel, A. J. Bard, Electrochemistry and electrogenerated chemiluminescence from silicon nanocrystal quantum dots. *Science* **296**, 1293–1297 (2002).

17. P. P. Jha, P. Guyot-Sionnest, Electrochemical switching of the photoluminescence of single quantum dots. *J. Phys. Chem. C* **114**, 21138–21141 (2010).

18. W. Qin, R. A. Shah, P. Guyot-Sionnest, CdSeS/ZnS alloyed nanocrystal lifetime and blinking studies under electrochemical control. *ACS Nano* **6**, 912–918 (2011).

19. J. D. Rinehart, A. M. Schimpf, A. L. Weaver, A. W. Cohn, D. R. Gamelin, Photochemical electronic doping of colloidal CdSe nanocrystals. *J. Am. Chem. Soc.* **135**, 18782–18785 (2013).

20. K. Wu, Y.-S. Park, J. Lim, V. I. Klimov, Towards zero-threshold optical gain using charged semiconductor quantum dots. *Nat. Nanotechnol.* **12**, 1140–1147 (2017).

21. M. J. Fernée, C. Sinito, Y. Louyer, C. Potzner, T.-L. Nguyen, P. Mulvaney, P. Tamarat, B. Lounis, Magneto-optical properties of trions in non-blinking charged nanocrystals reveal an acoustic phonon bottleneck. *Nat. Commun.* **3**, 1287 (2012).

22. Y.-S. Park, W. K. Bae, J. M. Pietryga, V. I. Klimov, Auger recombination of biexcitons and negative and positive trions in individual quantum dots. *ACS Nano* **8**, 7288–7296 (2014).

23. A. L. Roest, J. J. Kelly, D. Vanmaekelbergh, E. A. Meulenkamp, Staircase in the electron mobility of a ZnO quantum dot assembly due to shell filling. *Phys. Rev. Lett.* **89**, 036801 (2002).

24. L. Jin, L. Shang, J. Zhai, J. Li, S. Dong, Fluorescence spectroelectrochemistry of multilayer film assembled CdTe quantum dots controlled by applied potential in aqueous solution. *J. Phys. Chem. C* **114**, 803–807 (2009).

25. B. L. Wehrenberg, D. Yu, J. Ma, P. Guyot-Sionnest, Conduction in charged PbSe nanocrystal films. *J. Phys. Chem. B* **109**, 20192–20199 (2005).

26. S. C. Boehme, D. Vanmaekelbergh, W. H. Evers, L. D. A. Siebbeles, A. J. Houtepen, In situ spectroelectrochemical determination of energy levels and energy level offsets in quantum-dot heterojunctions. *J. Phys. Chem. C* **120**, 5164–5173 (2016).

27. P. Guyot-Sionnest, C. Wang, Fast voltammetric and electrochromic response of semiconductor nanocrystal thin films. *J. Phys. Chem. B* **107**, 7355–7359 (2003).

28. S. C. Boehme, H. Wang, L. D. A. Siebbeles, D. Vanmaekelbergh, A. J. Houtepen, Electrochemical charging of CdSe quantum dot films: Dependence on void size and counterion proximity. *ACS Nano* **7**, 2500–2508 (2013).

29. D. Spittel, J. Poppe, C. Meerbach, C. Ziegler, S. G. Hickey, A. Eychmüller, Absolute energy level positions in CdSe nanostructures from potential-modulated absorption spectroscopy (EMAS). *ACS Nano* **11**, 12174–12184 (2017).

30. C. Galland, Y. Ghosh, A. Steinbrück, J. A. Hollingsworth, H. Htoon, V. I. Klimov, Lifetime blinking in nonblinking nanocrystal quantum dots. *Nat. Commun.* **3**, 908 (2012).

31. S. Sampat, N. S. Karan, T. Guo, H. Htoon, J. A. Hollingsworth, A. V. Malko, Multistate blinking and scaling of recombination rates in individual silica-coated CdSe/CdS nanocrystals. *ACS Photonics* **2**, 1505–1512 (2015).

32. R. Vaxenburg, A. Rodina, E. Lifshitz, A. L. Efros, Biexciton Auger recombination in CdSe/CdS core/shell semiconductor nanocrystals. *Nano Lett.* **16**, 2503–2511 (2016).

33. J. P. Philbin, E. Rabani, Electron–hole correlations govern auger recombination in nanostructures. *Nano Lett.* **18**, 7889–7895 (2018).

34. M. Manceau, S. Vezzoli, Q. Glorieux, F. Pisanello, E. Giacobino, L. Carbone, M. De Vittorio, A. Bramati, Effect of charging on CdSe/CdS dot-in-rods single-photon emission. *Phys. Rev. B* **18**, 035311 (2014).

35. A. K. Gooding, D. E. Gómez, P. Mulvaney, The effects of electron and hole injection on the photoluminescence of CdSe/CdS/ZnS nanocrystal monolayers. *ACS Nano* **2**, 669–676 (2008).

36. J. I. Climente, J. L. Movilla, J. Planelles, Auger recombination suppression in nanocrystals with asymmetric electron–hole confinement. *Small* **8**, 754–759 (2012).

37. A. Puntambekar, Q. Wang, L. Miller, N. Smieszek, V. Chakrapani, Electrochemical charging of CdSe quantum dots: Effects of adsorption *versus* intercalation. *ACS Nano* **10**, 10988–10999 (2016).

38. I. du Fossé, S. ten Brinck, I. Infante, A. J. Houtepen, Role of surface reduction in the formation of traps in *n*-doped II–VI semiconductor nanocrystals: How to charge without reducing the surface. *Chem. Mater.* **31**, 4575–4583 (2019).

39. M. Widmann, M. Niethammer, D. Y. Fedyanin, I. A. Khramtsov, T. Rendler, I. D. Booker, J. Ul Hassan, N. Morioka, Y.-C. Chen, I. G. Ivanov, N. T. Son, T. Ohshima, M. Bockstedte, A. Gali, C. Bonato, S.-Y. Lee, J. Wrachtrup, Electrical charge state manipulation of single silicon vacancies in a silicon carbide quantum optoelectronic device. *Nano Lett.* **19**, 7173–7180 (2019).

40. N. Mizuochi, T. Makino, H. Kato, D. Takeuchi, M. Ogura, H. Okushi, M. Nothaft, P. Neumann, A. Gali, F. Jelezko, J. Wrachtrup, S. Yamasaki, Electrically driven single-photon source at room temperature in diamond. *Nat. Photonics* **6**, 299–303 (2012).

41. P. Senellart, G. Solomon, A. White, High-performance semiconductor quantum-dot single-photon sources. *Nat. Nanotechnol.* **12**, 1026–1039 (2017).

42. N. Accanto, P. M. de Roque, M. Galvan-Sosa, S. Christodoulou, I. Moreels, N. F. van Hulst, Rapid and robust control of single quantum dots. *Light Sci. Appl.* **6**, e16239 (2016).

43. R. Meng, H. Qin, Y. Niu, W. Fang, S. Yang, X. Lin, H. Cao, J. Ma, W. Lin, L. Tong, X. Peng, Charging and discharging channels in photoluminescence intermittency of single colloidal CdSe/CdS core/shell quantum dot. *J. Phys. Chem. Lett.* **7**, 5176–5182 (2016).

44. X. Hou, J. Kang, H. Qin, X. Chen, J. Ma, J. Zhou, L. Chen, L. Wang, L.-W. Wang, X. Peng, Engineering Auger recombination in colloidal quantum dots via dielectric screening. *Nat. Commun.* **10**, 1750 (2019).

45. D. T. Sawyer, A. J. Sobkowiak, J. Roberts Jr., *Electrochemistry for Chemists* (John Wiley & Sons, ed. 2, 1995).
